# Preterm Birth Prevention: Effects of Vaginal Progesterone Administration on Blood Flow Impedance in Uterine-Fetal Circulation by Doppler Sonography

**DOI:** 10.5539/gjhs.v8n7p172

**Published:** 2015-11-18

**Authors:** Homeira Vafaei, Tarlan Zamanpour, Hadi Raeisi Shahraki

**Affiliations:** 1 Assistant professor of Perinatalogy, Maternal-Fetal Medicine Research Center, Perinatalogy Ward, Shiraz University of Medical Sciences, Shiraz, Iran; 2 Maternal-Fetal Medicine Research Center, Fellowship Perinatology Ward, Shiraz University of Medical Sciences, Shiraz, Iran; 3 Department of Biostatistics, School of Medicine, Shiraz University of Medical Sciences, Shiraz, Iran

**Keywords:** Doppler sonography, preterm birth, vaginal progesterone, vasodilatory effects

## Abstract

**Objective:**

The present study aimed to evaluate the effect of vaginal progesterone administration on maternal and fetal circulation to prevent preterm birth.

**Methods:**

The present prospective study was conducted on 35 women with singleton pregnancy at 18–33 weeks of gestation, who presented with at least one episode of preterm labor or asymptomatic short cervix, or past medical history of preterm birth. Doppler flow and Pulsatility Index (PI) assessment of the umbilical artery, fetal middle cerebral artery, uterine arteries, and ductusvenosus were performed before and 72 h after vaginal progesterone administration.

**Results:**

Results showed a significant reduction in the PI of the uterine artery following progesterone administration. Nevertheless, no significant changes were observed in the PI of other vessels. No significant difference was found in Doppler flow parameters in any of the examined vessels before or after progesterone treatment in women with Preterm Labor Pain (PLP). Yet, a statistically significant association was observed between short cervix complication in the current pregnancy and medical history of PLP in the previous pregnancy.

**Conclusion:**

Treatment with vaginal progesterone reduced the PI in the uterine arteries in the second and third trimesters of pregnancy. Thus, this medication may have useful vasodilatory effects on uterine-fetal vessels.

## 1. Introduction

Preterm birth is one of the most important issues among women around the world. It is in fact one of the most important causes of perinatal morbidity and mortality in pregnant women (Barda et al., 2010; Dodd et al., 2012; King et al., 2003). There is evidence that progesterone may play a role in preventing preterm birth (Barda et al., 2010; Dodd et al., 2012; Di Renzo et al., 2012). Administration of progesterone, including vaginal administration of progesterone gel or progesterone tablets (De Franco et al., 2007; Fonseca et al., 2007; Obrien et al., 2007) and intramuscular injection of 17 alpha-hydroxyprogesteronecaproate (Meis et al., 2003), for preventing preterm birth has been investigated in various studies. Some reports have shown that progesterone could prevent preterm birth in women with short cervix (as a sonographic finding) in the second trimester (Barda et al., 2010; De Franco et al., 2012; Fonseca et al., 2007).

The definitive mechanisms involved in the role of progesterone inpreventing preterm birth are not known yet. Nonetheless, several mechanisms described for the role of progesterone include anti-inflammatory effect, inhibition of gap-junction formation in the myometrium, and direct effect on the cervix (Romero, 2007). By applying vasodilatory effects on the vessels of fetus and uterus, progesterone might be effective in the prevention of preterm birth. The vasodilatory effects of progesterone in non-pregnant women (Deichert et al., 1969) and in the first trimester of pregnancy (Czajkowski et al., 2007; Habara et al., 2002) have been evaluated. Furthermore, several attempts have indicated that ultrasound evaluation of uterine and fetal arteries in pregnant women in the second trimester of pregnancy can be predictors of birth complications (Axt-Fliedner et al., 2005; Cruz-Martinez et al., 2009; Li et al., 2005). However, far too few studies have focused on the possible effects of progesterone on maternal or fetal circulation in late pregnancy, especially in high-risk women (such as those with short cervix). In 2010, Barda et al. published a paper in which they suggested a statistically significant decrease in the fetal Middle Cerebral Artery (MCA) Pulsatility Index (PI) with no effects on other fetal and uterine vessels flow following progesterone treatment. Moreover, no significant changes were observed in Doppler flow parameters in any of the vessels examined in these patients either before or after progesterone treatment. Besides, Borna et al.(2014) concluded that administeration of vaginal progesterone suppository led to a reduction in fetal MCA-PI and umbilical artery PI. The present study aimed to evaluate the effect of vaginal progesterone on uterine artery and fetal vessels by Doppler sonography among women in the second and third trimesters of pregnancy who were at risk of preterm birth.

## 2. Patients and Methods

The present prospective study was conducted in the Obstetrics and Gynecology Department of Hafez hospital, Shiraz, Iran. Written informed consents were obtained from all the participants and the study protocol was approved by the Ethics Committee of Shiraz University of Medical Sciences.

The study participants included 35 women of all women (nearly 500) with singleton pregnancy at 18–33 weeks of gestation who had been visited in the outpatient obstetrics clinic from January to December 2014. The inclusion criteria of the study were having experienced regular uterine contractions leading to at least one episode of preterm labor, change in cervical dilatation and effacement detected by vaginal examination, asymptomatic short cervix (less than 25 mm) detected incidentally by abdominal ultrasound in the second trimester that confirmed by transvaginal sonography, and also those having past medical history of preterm birth. Women who had received other tocolytic drugs, such as MgSO_4_, non-steroidal anti-inflammatory drugs, and calcium channel blockers accompanying with progesterone, were excluded from the study.

A complete Doppler flow assessment (Note 1) of the maternal and fetal circulation equipped with a 2-5MHz convex abdominal transducer with color imaging capabilities was used. This was conducted by a perinatalogist in the clinic and was blinded to the treatment. The patients were treated with 400-mg vaginal tablets (Cyclogest; Actavis UK, Devon, UK) by an independent gynecologist after a while from the first Doppler flow assessment. The Doppler flow assessment was repeated 72 hours after the first assessment.

Doppler signals were obtained from a free loop of umbilical cord and the number of waveforms obtained was at least 3 to 5 cycles. We evaluated the wave forms that were regular when the fetus was not breathing and not moving to obtain an accurate assessment of the umblical artery flow impedance.

The MCA doppler assessment is optimally performed in an axial section of the fetal brain moving toward the base of the skull. The MCA can be visualized even without Doppler, but color flow aids in sampling. It is preferable to sample the MCA in its proximal segment, approximately 2 mm from the origin of the artery.

Doppler interrogation of the DV was done when the fetus was quiet and was not breathing. The sample volume was set at the origin of the DV from the umbilical vein. This was where color Doppler demonstrates the highest blood velocity. The angle of insonation should be less than or equal to 30 degrees.

Doppler study of uterine arteriers was performed by directing the transducer toward the external iliac artery to view it in a longitudinal section, and then mapping of uterine artery was done by color Doppler as it crosses the external iliac artery.

All the patients underwent Doppler sonography and PI of the umbilical artery blood flow, middle cerebral artery, mean of uterine arteries, and DuctusVenosus were evaluated.

The continued monitoring of the patients was performed in Hafez high risk pregnancy clinic until the time of their parturitions.

### 2.1 Statistical Analysis

The data are presented as mean and Standard Deviation (SD). Paired T-test was used to compare the two dependent parametric continuous variables, while Wilcoxon signed rank test was used to compare the two dependent non-parametric continuous variables. To assess the assumption of independence between the two qualitative variables, Fisher’s exact test was used. All the analyses were performed using the SPSS statistical software version 15 and the figures were plotted using Prism software (version 3.0, GraphPad Software, San Diego, Calif). P<0.05 was considered as statistically significant.

## 3. Results

The present study was performed for 35 patients aged 20- 34 years old (27.06 (6.33) years). The patients had singleton pregnancies at 18–33 weeks of gestation (28.79 (4.13 weeks).

**Figure 1:**
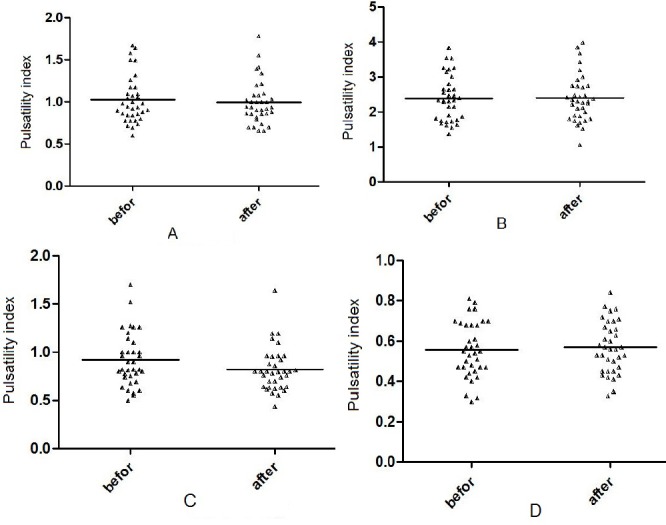
Correlation of pulsatility index in the umbilical artery (A), fetal middle cerebral artery (B), uterine artery (C) and ductusvenosus (D) before and after maternal progesterone treatment

On ultrasound examination at admission, 13 participants (37.1%) were found to have short cervix; i.e., cervical length ≤25 mm. The results revealed no significant changes in Doppler flow parameters assessment of all the examined blood vessels (maternal uterine artery, umbilical artery, MCA, and DV) in the patients with and without short cervix before and after the treatment (P>0.05). Also, no significant differences were observed between patients with and without short cervix regarding gestational age (29.74(2.98) and 28.23(4.66) weeks, respectively) and age (26.08(6.66) and 27.63(6.21) years, respectively). The results showed a significant decrease in the PI of the maternal uterine artery following progesterone administration (mean reduction in PI, 0.1 (10.8%), P=0.001). Nevertheless, no significant changes were observed in the Doppler flow parameters of the other blood vessels studied (umbilical artery, MCA, and ductusvenosus) (Figure 1) (Table 1).

**Table 1:** Doppler flow parameters of maternal and fetal circulation before and after progesterone treatment

Parameter	Before progesterone treatment	After progesterone treatment	P-value
**PI umbilical**	1.02 (0.28)	0.99 (0.26)	0.41
**PI MCA**	2.39 (0.65)	2.39 (0.67)	0.97
**Mean PI uterine artery**	0.92 (0.28)	0.82 (0.23)	0.001
**PI DV**	0.56 (0.14)	0.57 (0.13)	0.68

*MCA* fetal middle cerebral artery, *PI* pulsatility index, *DV*DuctusVenosus

No significant difference was found in Doppler flow parameters in any of the examined vessels before or after progesterone treatment in women with Preterm Labor Pain (PLP) compared with those without PLP.Yet, woman with a medical history of PLP in the previous pregnancy presented a high proportion (77.8%) of short cervix complication in the current pregnancy, and this association was statistically significant (P=0.006). The patients’ characteristics based on PLP are presented in Table 2.

**Table 2:** Preterm labor pain in women treated with progesterone to prevent preterm birth

Characteristics	Number (%)	Gestational age (weeks)	P-value	Age (years)	P- value
With PLP in the current pregnancy	9 (25.7)	30.38 (3.15)*		28.44 (9.23)	
without PLP in the current pregnancy	26 (74.3)	28.23 (4.34)	0.12	26.58 (5.12)	0.6
Positive history of PLP	6 (17.1)	27.47 (3.59)		30.83 (6.37)	
**Negative history of PLP**	**29 (82.9)**	**29.06 (4.24)**	0.3	**26.28 (6.07)**	0.11

* Data are presented as mean (SD), *PLP* preterm labor pain.

## 4. Discussion

Progesterone is a steroid hormone produced by the adrenals, gonads, nervous system, and placenta in pregnancy. The vaginal route of progesterone has been termed as the ‘first uterine pass effect’, suggesting direct transit of vaginal progesterone into the uterus (Cicinelli et al., 2000; Fanchin et al., 1997). Rapid absorption, avoiding first-pass hepatic metabolism, and high bioavailability especially locally in the most desired target organ, the uterus, are the advantages of this form (Khandelwal, 2012). The useful role of vaginal progesterone in the women with the risk of preterm labor was met with controversial findings (Khandelwal, 2012). For instance, a systematic review and meta-analysis by Romero et al. (2012) demonstrated that vaginal progesterone administration to asymptomatic women with sonographicshort cervix reduced the risk of preterm birth and neonatal morbidity and mortality. On the other hand, the results of a multi-centered, randomized, double-blind, placebo-controlled trial in 2014 indicated that there was no evidence in favor of decrease in preterm birth or improvement in neonatal outcomes in women with preterm labor (24-33 weeks of gestation) following daily administration of 200 mg vaginal progesterone (Martinez et al., 2015).

By applying vasodilatory effects on the vessels of fetus and uterus, progesteronemight be effective in prevention of preterm birth. The vasodilatory effect of progesterone has been demonstrated previously in non-pregnant women as well as in the first trimester of pregnancy (Deichert et al., 1969; Czajkowski et al., 2007; Habara et al., 2002). The possible explanations for this effect are its non-genomic effect (Romero, 2007) and regulation of vascular tone by induced rapid inhibition of platelet aggregation (Bar et al., 2000). Nonetheless, Czajkowski et al. (2007) demonstrated that vaginal progesterone, different from oral dydrogesterone, led to a slow decrease in spiral artery PI of uncomplicated pregnancy, which was attributable to increasing progesterone levels (Makikallio et al., 2004). Hence, the definitive mechanisms involved in vasodilatory effect of progesterone remain unknown.

According to these discrepant findings, we aimed to use 400 mg vaginal progesterone to evaluate the effect of vaginal progesterone on uterine artery and fetal vessels by Doppler sonography in women in the second and third trimesters of pregnancy who were at risk of preterm birth.

In 2010, Barda et al. demonstrated a statistically significant decrease in fetal MCA-PI 24 h after progesterone administration (mean reduction of 18.2%, mean PI change of 0.44, *P*<0.001). However, they detected no significant changes in fetal MCA-PSV and the PI of the uterinearteries and umbilical artery. The findings of our study were inconsistent with those obtained by Barda et al. which might be due to the fact that some of the women in the study by Barda et al. (2010) were treated with fluids, tocolytic agents, non-steroidal anti-inflammatory agents, or steroids before the study and the impacts of probable interactions of these drugs on their results were not eliminated.

In the study by Borna (2014), fetal Doppler velocimetry was investigated before and 24 hours and two weeks after administration of vaginal progesterone in 30 patients with intrauterine growth retardation and 30 pregnant women with threatened preterm labor. They showed that the use of vaginal progesterone suppository led to a reduction in MCA PI after 24 hours and a reduction in PI of umbilical artery after two weeks of treatment.

In the two recent studies, fetal MCA-PI was reduced 24h after treatment. Nevertheless, our findings showed no significant decrease in fetal MCA-PI 72h after the treatment. This inconsistency may be due to differences in the time of Doppler flow parameters assessment after treatment. Furthermore, the study by Borna (2014) demonstrated no significant reduction in PI, systolic/diastolic ratio, and resistance index (RI) of umbilical artery after 24 hours, whereas the reduction in PI was significant after two weeks of treatment. A possible explanation for these differences may be the length of treatment. Yet, more research on this topic is required to determine the association between time of assessment and length of treatment and Doppler flow parameters.

One of the limitations of this study was the wide gestational age range (18–33 weeks), which might have presented possible confounders, such as different responses of various blood vessels. Furthermore, we do not complete the comparison between women who may have preterm birth and women who deliver at term, which was another limitation of this study.

In conclusion, our study demonstrated that vaginal administration of progesterone for prevention of preterm birth was associated with a significant decrease in the PI of the uterine artery. Yet, further studies are required to investigate the impact of progesterone’s vasodilatory effects on uterine-fetal circulation at different times and different treatment lengths.
